# The role of lncRNAs in regulation of DKD and diabetes-related cancer

**DOI:** 10.3389/fonc.2022.1035487

**Published:** 2022-10-13

**Authors:** Yawei Cheng, Xiaowen Wu, Yujie Xia, Wenjun Liu, Peter Wang

**Affiliations:** ^1^ Department of Disease Prevention, Hainan Province Hospital of Traditional Chinese Medicine, Haikou, China; ^2^ Hainan Clinical Research Center for Preventive Treatment of Diseases, Haikou, China; ^3^ Department of Food Science and Technology Centers, National University of Singapore (Suzhou) Research Institute, Suzhou, China; ^4^ Department of Research and Development, Zhejiang Zhongwei Medical Research Center, Hangzhou, China

**Keywords:** lncRNAs, cancer, diabetes, DKD, miRNAs, treatment

## Abstract

Diabetes mellitus often results in several complications, such as diabetic kidney disease (DKD) and end-stage renal diseases (ESRDs). Cancer patients often have the dysregulated glucose metabolism. Abnormal glucose metabolism can enhance the tumor malignant progression. Recently, lncRNAs have been reported to regulate the key proteins and signaling pathways in DKD development and progression and in cancer patients with diabetes. In this review article, we elaborate the evidence to support the function of lncRNAs in development of DKD and diabetes-associated cancer. Moreover, we envisage that lncRNAs could be diagnosis and prognosis biomarkers for DKD and cancer patients with diabetes. Furthermore, we delineated that targeting lncRNAs might be an alternative approach for treating DKD and cancer with dysregulated glucose metabolism.

## Introduction

Noncoding RNAs have been known to play an essential role in development of many diseases (1, 2). Noncoding RNAs include short non-coding RNAs, such as microRNAs (miRNAs), small interfering RNA (siRNAs), piwi-interacting RNA (piRNAs), transfer RNA (tRNAs), small nuclear RNA (snRNAs) and small nucleolar RNA (snoRNAs), and long non-coding RNAs (lncRNAs), which is based on their length (3). LncRNAs often have more than 200 nucleotides and cancer serve as signal molecules, decoy molecules, guide molecules, and scaffold molecules to perform their functions *via* regulation of gene expression at epigenetic, transcriptional and post-transcriptional levels (4, 5). Accumulated evidence has dissected that lncRNAs participate in cellular biological processes *via* regulation of protein degradation and governing gene transcription as well as controlling protein coding sections (6–8). Dysregulated lncRNAs have been reported to participate in numerous diseases, including cancer, inflammatory bowel disease, cardiovascular disease, neurological disorders and diabetes (9–14).

Diabetes mellitus (DM) has become a major health problem in the world, which often results in several complications, such as diabetic kidney disease (DKD) (15). DKD is often known as diabetic nephropathy. DM has three types: type 1 diabetes, type 2 diabetes and gestational diabetes (GDM). Type 1 diabetes is insulin-dependent and often appears during childhood and adolescence. Type 2 diabetes often appears in older adults due to that pancreas does not make enough insulin or cells respond poorly to insulin. GDM often happens during the pregnancy after insulin secretion is not enough. DKD is one of causes to develop end stage kidney disease (ESKD) and kidney failure (16). It has been known that chronic stimuli such as high glucose in the bloodstream can lead to pathological gene modulation and DKD in diabetic patients (17). EMT and endothelial-mesenchymal transition (EndMT) have been characterized to integrate into the fibrosis and DKD (18, 19). EMT is a process in which epithelial cells acquire mesenchymal characteristics after various stimulations. Similarly, EndMT is a process in which endothelial cells have the phenotype toward mesenchymal cells, which often appears in cardiovascular diseases. Cancer patients often have the dysregulated glucose metabolism. Abnormal glucose metabolism can enhance the tumor malignant progression (20).

Recently, noncoding RNAs, including lncRNAs, have been reported to regulate the key proteins and signaling pathways in DM and DKD development and progression as well as in cancer with diabetes (21–24). In this review article, we elaborate the evidence to support the function of lncRNAs in development of DKD and cancer patients with diabetes. Moreover, we envisage that lncRNAs could be diagnostic and prognosis biomarkers for DKD and diabetes-related cancers. Furthermore, we delineated that targeting lncRNAs might be an alternative approach for treating DKD and diabetes-associated cancer.

## Role of lncRNAs in DKD

Emerging evidence has suggested that lncRNAs are useful for precision medicine in DKD (25–28). Zhang and colleagues used the integrate biological, computational, and statistical strategies to analyze the pathogenesis and progression of DKD through analysis of regulatory networks including miRNAs, lncRNAs and mRNAs (29). This study reported that 127 lncRNAs were changes in DKD, among which 26 were decreased and 101 were increased. In particular, this work identified that miR-223-3p might be a biomarker for prediction of DKD disease process (29).

### LncRNA HOTAIR

Evidence showed that lncRNA HOTAIR is critically involved in DKD development (30). One group used several mouse models, such as podocyte-specific Hotair knockout mice, streptozotocin-induced diabetes in mice, and the db/db mouse model of type 2 diabetes. In these mouse models, glomerular HOTAIR was upregulated. Depletion of Hotair in podocytes did not affect structure, ultrastructure, function of kidneys (30). In mouse podocytes, high glucose treatment increased the expression of HOTAIR. Interestingly, silencing of HOTAIR did not affect the kidney damage in diabetic mice. Moreover, HOTAIR expression was linked to HOXC11 expression in human kidney tissues according to a bioinformatic assay (30). Notably, the serum level of HOTAIR was increased in type 2 DM patients (31). HOTAIR can be a useful biomarker in prediction of diabetic retinopathy and DKD in patients with type 2 DM. In addition, HOTAIR facilitated high glucose-mediated fibrosis and proliferation of mesangial cells *via* affecting miR-147a/WNT2B axis in diabetic nephropathy (32). The role of HOTAIR in DKD needs to be ascribed to validate its function in the pathogenesis of DKD.

### LncRNA GAS5

Wang et al. reported that lncRNA GAS5 promoted renal tubular epithelial fibrosis *via* sponging miR-96-5p (33). Renal fibrosis is often observed in DKD. Higher expression of lncRNA GAS5 was reported in renal proximal tubular cells after TGF-β1 treatment. The kidneys of high-fat diet (HFD)/streptozotocin (STZ) mice had the upregulation of lncRNA GAS5 (33). Silencing of lncRNA GAS5 reduced renal fibrosis *via* inhibition of miR-96-5p. Consistently, DKD mice had the lower expression of miR-96-5p, leading to upregulation of fibronectin. Hence, depletion of lncRNA GAS5 could have antifibrosis *via* sponging miR-96-5p and regulating fibronectin. Zhang et al. found that lncRNA GAS5 attenuated TGF-β-mediated renal fibrosis by inhibition of collagen type 1 an fibronectin *via* targeting the Smad3/miR-142-5p axis (34). LncRNA GAS5 suppressed fibrosis and cell proliferation through attenuating miR-221 and upregulating SIRT1 expression in diabetic nephropathy (35). LncRNA GAS5 inhibited pyroptosis and oxidative stress in renal tubular cells after high glucose stimulation (36). LncRNA GAS5 alleviated fibrosis *via* inhibition of MMP9 by recruitment of EZH2 in diabetic nephropathy (37). Altogether, modulation of lncRNA GAS5 might be useful for preventing DKD.

### LncRNA MALAT1

LncRNA MALAT1 has been identified to play key roles in DKD pathophysiology (38). One work assessed urinary albumin in 136 patients with type 2 DM and 25 normal people. This work found that urinary lncRNA MALAT1 was positively associated with urinary podocalyxin, synaptopodin, UACR (urinary albumin), NAG (N-acetyl-D-glucosaminidase), KIM-1 (kidney injury molecule 1), miR-21, miR-93, miR-29a (38). LncRNA MALAT1 was negatively correlated with eGFR, miR-29a and miR-93. In addition, urinary lncRNA MIAT was positively linked to miR-29a, miR-93 and eGFR, while lncRNA MIAT was negatively associated with miR-21, miR-124, UACR, NAG and KIM-1 (38). In line with this report, the expression of lncRNA MALAT1 in PBMC was increased in type 2 DM and DKD (39). MALAT1 was associated with ACR, HbA1c, SOD, creatinine, α1-MG and β2-MG in type 2 DM and DKD patients. MALAT1 in combination with ACR, α1-MG and creatinine could be helpful for prediction of DKD in DM patients (39). MALAT1 enhanced diabetic nephropathy *via* suppression of miR-15b-5p and upregulation of TLR4 signaling (40).

MALAT1 activated LIN28 and Nox4/AMPK/mTOR pathway, resulting in promotion of renal tubular injury in diabetic nephropathy (41). Huang et al. reported that MALAT1 aggravated renal fibrosis *via* modulation of miR-2355-3p/IL6ST axis in diabetic nephropathy (42). One study showed that podocyte injury could be due to abnormal MALAT1 expression and subsequent dysregulated let-7f and KLF5 in diabetic nephropathy (43). MALAT1 was also reported to participate in high glucose-mediated HK-2 cell EMT *via* activation of Wnt/β-catenin pathway and injury (44). Consistently, MALAT1 was involved in high glucose-mediated podocyte injury in diabetic nephropathy *via* its interaction with β-catenin (45). MALAT1 aggravated high glucose-triggered EndMT and fibrosis through regulation of miR-145/ZEB2 axis (46). Additionally, MALAT1 participated in high glucose-mediated HK-2 cell injury *via* interplay with Foxo1 to affect SIRT expression (47).

### LncRNA MIAT

Urinary lncRNA MIAT was positively linked to miR-29a, miR-93 and eGFR, while lncRNA MIAT was negatively associated with miR-21, miR-124, UACR, NAG and KIM-1 in type 2 DM (38). Depletion of lncRNA MIAT mitigated apoptosis and inflammation in podocyte after high glycose stimulation through modulating miR-130a-3p and TLR4 pathway (48). Ablation of lncRNA MIAT ameliorated fibrosis and cell proliferation *via* suppression of E2F3 expression in diabetic nephropathy (49). Loss of lncRNA MIAT blocked podocyte injury and mitotic damage in diabetic nephropathy (50). However, one study showed that lncRNA MIAT blocked the high glucose-mediated cell damage and activation of NF-κB *via* sponging miR-182-5p and elevating the GPRC5A expression in diabetic nephropathy, leading to suppression of diabetic nephropathy progression (51).

### LncRNA NEAT1

Evidence has suggested that lncRNA NEAT1 governed renal tubular EMT *via* regulation of the ERK1/2 signaling pathway in DKD (52). LncRNA NEAT1 was increased in BSA-treated HK2 cells and HFD/STZ-induced DKD mice. Depletion of NEAT1 suppressed the expression of the EMT-related markers, such as vimentin and a-SMA, and the renal fibrosis-associated markers, including TGF-β1 and CTGF (52). LncRNA NEAT1 regulated DKD progression *via* modulation of the ERK1/2 signaling pathway. Li et al. discovered that NEAT1 interacted with miR-129 to promote renal fibrosis *via* upregulation of collagen type 1 and promotion of EMT process (53). Additionally, urinary lncRNA NEAT1 was positively correlated with miR-21, miR-124, KIM-1, synaptopodin, and NAG in type 2 DM. Urinary lncRNA NEAT1 had a negative association with miR-29a, miR-93 and eGFR (38).

LncRNA NEAT1 activated Akt/mTOR pathway and accelerated cell fibrosis and proliferation in diabetic nephropathy (54). LncRNA NEAT1 enhanced EMT and accumulation of extracellular matrix in diabetic nephropathy *via* sponging miR-27b-3p and ZEB1 (55). Ablation of lncRNA NEAT1 attenuated proliferation, fibrosis and inflammation of mouse mesangial cells in diabetic nephropathy (56). In addition, lncRNA NEAT1 accelerated diabetic nephropathy occurrence and progression *via* suppression of miR-23c (57). LncRNA NEAT1 affected pyroptosis *via* targeting the miR-34c and NLRP3 in diabetic nephropathy (58). One group showed that lncRNA NEAT1 accelerated high glucose-triggered hypertrophy in mesangial cells through modulating miR-222-3p and CDKN1B (59). Yang et al. found that lncRNA NEAT1 enhanced tubular epithelial cell damage in kidney through regulation of mitophagy by targeting miR-150-5p and DRP1 in diabetic nephropathy (60). Moreover, lncRNA NEAT1 promoted fibrosis, inflammation, proliferation and oxidative stress by modification of the miR-423/5p and GLIPR2 pathway in diabetic nephropathy (61). Hence, lncRNA NEAT1 might be a promising therapeutic target for the treatment of DKD.

### LncRNA TUG1

LncRNA TUG1 has been identified to play a crucial role in DKD progression (62). One study revealed that ChREBP controlled lncRNA TUG1 transcription when glucose levels were increased in podocytes (62). Besides ChREBP, other coregulates, such as MXD1, MLX and HDAC1, were increased at the TUG1 promoter in response to high glucose exposures. This work suggested that ChREBP coordinated glucose homeostasis *via* regulation of lncRNA TUG1 (62). In addition, lncRNA TUG1 was discovered to regulate mitochondrial bioenergetics *via* regulation of PGC-1α in podocytes in diabetic nephropathy (63, 64). Overexpression of TUG1 in podocytes ameliorated diabetes-mediated chronic kidney disease in mice (63). Zhang et al. reported that knockdown of lncRNA TUG1 retarded the EMT of renal tubular epithelial cells *via* targeting miR-141-3p/β-catenin (65). Another work also demonstrated that lncRNA TUG1 reduced accumulation of extracellular matrix by sponging miR-377 and targeting PPARγ in diabetic nephropathy (66). Moreover, lncRNA TUG1 interacted with miR-9 and upregulated SIRT1, resulting in protection of podocytes from high glucose-triggered apoptosis and mitochondrial dysfunction (67). Urinary lncRNA TUG1 was positively associated with miR-29a, miR-93 and eGFR in type 2 DM, while lncRNA TUG1 had a negative association with miR-21, miR-124, podocalyxin, NAG and synaptopodin (38).

LncRNA TUG1 participated in regulation of podocyte apoptosis *via* modulation of TRAF5 pathway in diabetic nephropathy rats (68). LncRNA TUG1 influenced podocyte apoptosis *via* promotion of endoplasmic reticulum stress in diabetic nephropathy progression (69). Additionally, lncRNA TUG1 repressed the PI3K/AKT pathway and suppressed the fibrosis and proliferation in mesangial cells in diabetic nephropathy (70). LncRNA TUG1 inhibited the expression of miR-21 and enhanced the TIMP3 expression, leading to ameliorating diabetic nephropathy (71). LncRNA TUG1 repressed the PU.1/RTN1 pathway and improved diabetic nephropathy (72). Notably, lncRNA TUG1 affected high glucose-stimulated renal epithelial cell injury *via* regulation of endoplasmic reticulum stress by targeting miR-29c-3p and SIRT1 in diabetic nephropathy (73).

### LncRNA MEG3

LncRNA MEG3 has been revealed to regulate glucose metabolisms in diabetic mice (74). STZ-mediated diabetic mice had an increased expression of lncRNA MEG3, which was associated with the podocyte numbers. Mice with knockdown of MEG3 in podocyte had improved renal physiological and histopathological features (74). These mice also had a reduced mitochondrial translocation of Drp1 and a decreased podocyte damage (74). Overexpression of lncRNA MEG3 in podocyte led to podocyte injury and enhanced mitochondria damage and upregulated expression and phosphorylation of Drp1 (74). LncRNA MEG3 increased fibrosis and inflammation through regulating miR-181a, Egr-1 and TLR4 in diabetic nephropathy (75). Moreover, lncRNA MEG3 sponged miR-145 and impacted the development of diabetic nephropathy (76). Strikingly, lncRNA MEG3 inactivated the Wnt/β-catenin pathway and reduced podocyte injury in diabetic nephropathy (77). Therefore, MEG3 plays an essential role in diabetic mice and DKD.

### LncRNA KCNQ1OT1

Downregulation of KCNQ1OT1 attenuated oxidative stress and inflammation and reduced pyroptosis in renal tubular epithelial cells after high glucose stimulations through regulation of miR-506-3p (78). One study showed that KCNQ1OT1 participated in governing fibrosis, apoptosis and proliferation *via* regulation of miR-18b-5p and SORBS2 and NF-κB in diabetic nephropathy (79). Another study revealed that KCNQ1OT1 sponged miR-18b and increased the expression of HMGA2 and led to controlling high glucose-triggered oxidative stress, proliferation and extracellular matrix promotion in mesangial cells (80). In addition, KCNQ1OT1 was reported to accelerate diabetic nephropathy development *via* modulating miR-93-5p/ROCK2 axis (81). Xu et al. dissected that KCNQ1OT1 governed cell oxidative stress, proliferation, inflammation and extracellular matrix enhancement through miR-147a/SOX6 pathway in diabetic nephropathy (82). Recently, KCNQ1OT1 expression in diabetic nephropathy was increased and associated with activation of MEK/ERK pathway in diabetic nephropathy (83). LncRNA KCNQ1OT1 participates in DKD development and progression.

### LINC00472

Wang et al. used the data from Gene Expression Omnibus (GEO) database to explore the differentially expressed profiles between DKD patients and the normal patients. This study found that among 252 lncRNAs, 14 lncRNAs were differentially expressed. LINC00472 was identified to be differentially expressed in DKD patients, suggesting that LINC00472 could act as the diagnostic biomarkers for DKD patients (84). It is required to explore the detailed role of LINC00472 in DKD.

### LncRNA NONMMUG023520.2 and NONMMUG032975.2

Smad3 has been reported to enhance the development of type 2 DM and involve in DKD pathogenesis (85–87). One group discovered the Smad3-associated genes *via* analysis of whole transcriptome profile in three types of transgenic mouse models, including Smad3 WT-db/db, Smad3 KO-db/db, Smad3^+/-^ db/db mice (88). Smad3 KO-db/db mice displayed dysregulated genes involved in metabolism and RNA splicing, Smad3^+/-^ db/db mice exhibited dysregulated genes that were associated with cell cycle and cell division (88). Two lincRNAs, NONMMUG023520.2 and NONMMUG032975.2, were further validated to be linked to the pathogenesis of diabetic nephropathy. Moreover, Upk1b, Psca and Gdf15 were identified to be correlated with diabetic nephropathy development [26. Without a doubt, further investigation is pivotal to determine the function of lncRNA NONMMUG023520.2 and NONMMUG032975.2 in DKD development and pathogenesis.

### LncRNA 254693

Increased evidence has revealed that lncRNA ENSG00000254693 participated in DKD development (89). One research used RNA sequencing data and observed numerous differentially expressed lncRNAs in renal specimens of DKD. Among these dysregulated lncRNAs, lncRNA ENSG00000254693 was drastically changed. Moreover, DKD patients had higher expression of lncRNA ENSG00000254693 (89). Consistently, lncRNA ENSG00000254693 was upregulated in human podocytes after high glucose exposures. Depletion of lncRNA 254693 attenuated apoptosis, inflammation, and podocyte injury that were induced by high glucose (89). Furthermore, lncRNA 254693 was found to combine with HuR, and depletion of lncRNA 254693 reduced HuR levels. Interestingly, silencing of HuR reduced the expression and stability of lncRNA 254693 and alleviate podocyte injury, apoptosis and inflammation (89). Therefore, lncRNA 254693 might be a predicted factor for DKD treatment.

### Other lncRNAs in DM and DKD

LncRNA CASC2 expression in renal samples and serum was identified to be downregulated in type 2 DM patients with chronic renal failure (90). Low serum level of CASC2 was associated with higher incidence of kidney failure, indicating that serum lncRNA CASC2 could be a biomarker for prediction of the occurrence of kidney failure in type 2 DM patients (90). By RT-PCR analysis in 77 type 2 DM patients, 60 diabetic nephropathy and 60 healthy people, one group found that lncRNA PANDAR in the serum was upregulated compared with healthy people (91). PANDAR expression was linked to the level of proteinuria and glomerular filtration rate. PANDAR might serve as a biomarker for judgement of DKD prognosis (91). Yang et al. reported the differential expression profiles of circulating lncRNAs in DM and DKD patients. Compared with healthy persons, 245 lncRNAs were increased, while 680 lncRNAs were decreased in the serum of DM patients. Compared with diabetes patients, 45 and 813 lncRNAs were increased and decreased in the serum of DKD patients, respectively (92). LncRNA ARAP1-AS1 expression was elevated during DM and DKD progression, while lncRNA ARAP1-AS2 was decreased in DM and DKD progression (92). Hence, circulating lncRNA ARAP1-AS1 and ARAP1-AS2 might predict the progression of DM and DKD.

Another group identified that lncRNA KCNQ1OT1 was abnormally elevated in PBMCs of diabetic nephropathy, which was correlated with the activation of MEK/ERK pathway (83). LncRNA CASC2 modulated cell proliferation, oxidative stress and extracellular matrix promotion in human mesangial cells upon high glucose treatment through regulation of miR-133b and FOXP1 expressions (93). LncRNA CASC2 mitigated diabetic nephropathy development *via* sponging miR-144 and regulating SOCS2 expression (94). LncRNA CASC2 ablated cell inflammation, proliferation and fibrosis in glomerular mesangial cells upon high glucose exposures *via* targeting miR-135a-5p/TIMP3 pathway and JNK pathway (95).

## LncRNAs regulate glucose metabolism in cancer

**Figure 1:**
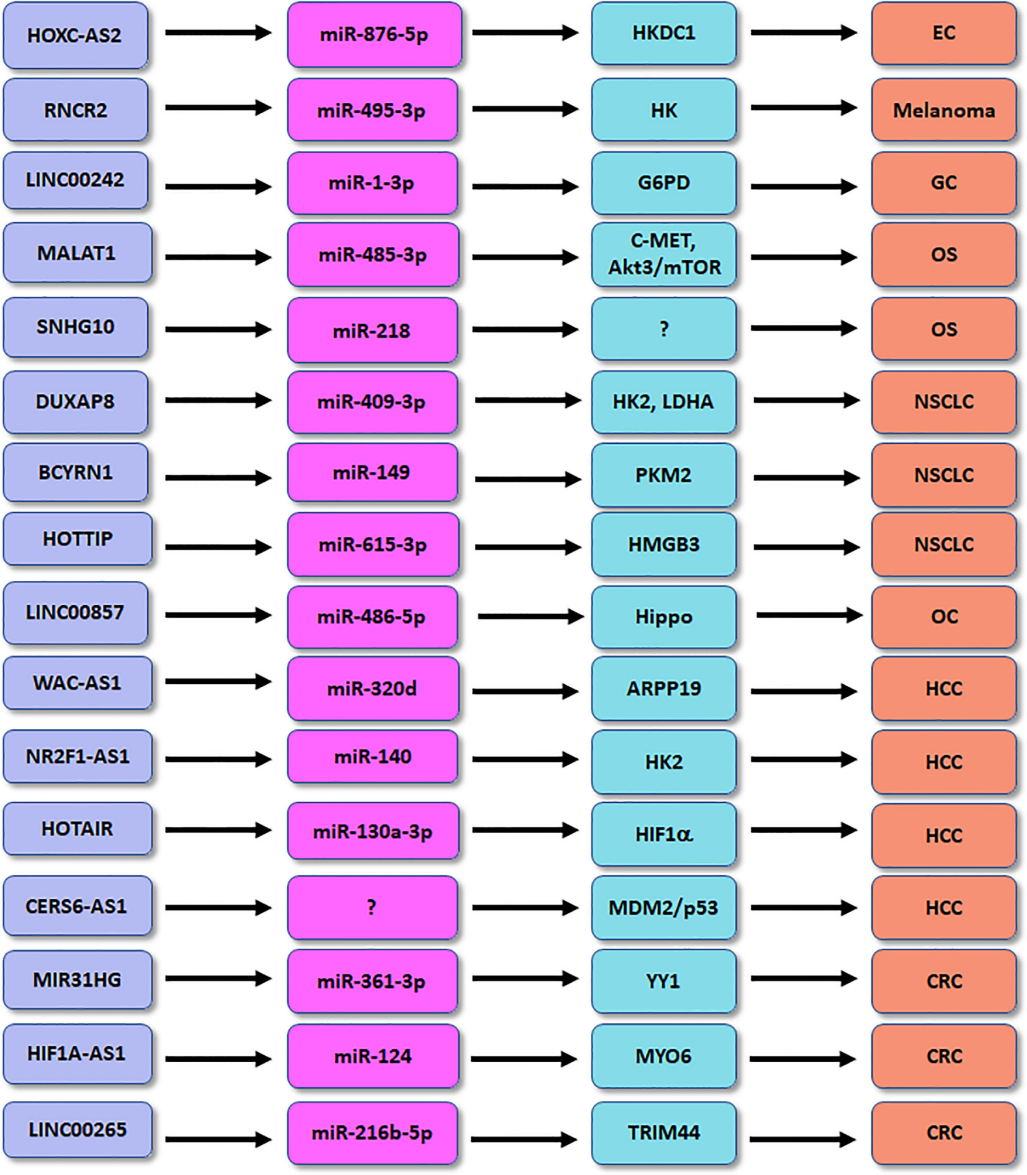
The role of lncRNAs in regulation of glucose metabolism in human cancers. EC, endometrial cancer; OS, osteosarcoma; HCC, hepatocellular carcinoma; CRC, colorectal cancer; GC, gastric cancer; NSCLC, non-small cell lung cancer.

Competing endogenous RNAs (ceRNA) can compete for shared miRNAs to modulate the expression of other RNA transcripts. A ceRNA network profile has identified the several lncRNAs for classifying diabetic pancreatic cancer form non-diabetic pancreatic cancer, including HOTAIR, CECR7, UCA1, suggesting that lncRNAs are important predictors for diabetic pancreatic cancer (96). In the following paragraphs, we will discuss the association between lncRNAs and glucose metabolisms in human cancer (**Figure 1**).

### LncRNAs regulate glucose metabolism in cancer

Evidence has dissected that lncRNA-associated genetic variants are shared between cancers and type 2 DM in human (97). LncRNA DRAIR has been known to involve in the development of type 2 DM (98). One study showed that the expression of lncRNA DRAIR was remarkably elevated in triple-negative breast cancer (TNBC) samples and plasma (99). High expression of DRAIR in plasma was associated with chemoresistance after therapy and tumor recurrence in TNBC patients. *In vitro* experiments showed that overexpression of DRAIR enhanced proliferation and viability of TNBC cells after doxorubicin treatment (99).

Accumulated evidence dissected that lncRNA HOXC-AS2 participated in the progression in high glucose-related endometrial cancer (EC) (100). EC patients with diabetes had the increased expression of HKDC1 compared with EC patients with normal glucose. HKDC1 governed pyroptosis, a highly inflammatory response of regulated cell death, *via* regulation of ROS and cytokine release in EC cells after high glucose stimulation (100). Moreover, miR-876-5p can inhibit the expression of HKDC1 in high glucose-related EC. LncRNA HOXC-AS2 was dissected to suppress the miR-876-5p/HKDC1 axis in high glucose-associated EC (100). HKDC1 affected the formation of TME *via* promotion of glycolysis, leading to accelerating EC progression. This work provided the new therapeutic strategy for EC patients with diabetes by targeting lncRNA HOXC-AS2 (100). LncRNA SNHG10 enhanced glucose uptake and increased proliferation of osteosarcoma cells *via* promotion of miR-218 methylation (101). LncRNA MALAT1 facilitated glycolysis and tumor metastasis *via* blocking miR-485-3p and upregulating c-MET and Akt3/mTOR pathways in osteosarcoma (102). LncRNA CERS6-AS1 regulated the MDM2/p53 axis and modulated glucose metabolism and progression of HCC (103). LncRNA WAC-AS1 sponged miR-320d and regulated the expression of ARPP19, which promoted glucose uptake and lactate production in HCC (104). LncRNA NR2F1-AS1 affected hypoxia-mediated glycolysis and migratory ability of HCC cells *via* targeting miR-140 and HK2 (105). Depletion of lncRNA HOTAIR reduced glycolysis *via* inhibition of miR-130a-3p and upregulation of HIF1α in HCC cells under hypoxia (106).

LncRNA MALAT1 modulated MYBL2/mTOR pathway and caused glucose metabolism changes in prostate cancer (107). LncRNA MIR31HG heightened glycolysis and tumor malignant progression *via* regulating miR-361-3p and YY1 transcription factor in colorectal cancer (108). LncRNA KCNQ1OT1 accelerated colorectal oncogenesis *via* promoting aerobic glycolysis by upregulation of HK2 (109). HNF1A-AS1 governed glycolysis, invasion and migration through targeting miR-124 and MYO6 in colorectal cancer (110). Similarly, LINC00265 enhanced glycolysis and lactate release *via* binding with miR-216b-5p and elevating the expression of TRIM44 in colorectal cancer (111). LncRNA RNCR2 promoted glycolysis and EMT and proliferation of melanoma cells *via* interacting with miR-495-3p and upregulating HK2 in melanoma (112). LINC00242 combined miR-1-3p and elevated the expression of G6PD, leading to enhancement of aerobic glycolysis and oncogenesis of gastric cancer (113). LncRNA MSC-AS1 increased glycolysis and cell growth *via* targeting PFKFB3 expression in gastric cancer cells (114). OIP5-AS1 heightened aerobic glycolysis and proliferation *via* miR-186 sponge in gastric cancer (115).

LINC00551 inhibited glycolysis and blocked tumor progression *via* modulation of c-Myc-induced PKM2 expression in lung cancer (116). LncRNA CRYBG3 potentiated glycolysis *via* interaction with lactate dehydrogenase A (LDHA) in lung cancer (117). LncRNA DUXAP8 accelerated glycolysis, viability and migratory capacities *via* suppression of miR-409-3p and upregulation of HK2 and LDHA in NSCLC cells (118). LncRNA BCYRN1 accelerated glycolysis *via* controlling the miR-149 expression and elevating PKM2 expression in NSCLC (119). HOTTIP enhanced hypoxia-mediated glycolysis *via* modulation of miR-615-3p and HMGB3 in NSCLC cells (120). LINC00857 was found to regulate glycolysis and tumor progression *via* governing the Hippo signaling pathway by binding to miR-486-5p in ovarian cancer (121). Downregulation of lncRNA UCA1 attenuated glycolysis pathway and led to suppression of growth of pituitary cancer cells (122). Overexpression of lncRNA PCED1B-AS1 resulted in upregulation of glucose uptake, proliferation and lactate production in glioblastoma by activation of HIF-1α pathway (123). LncRNA HNF4A-AS1 elevated aerobic glycolysis and tumor progression *via* modulating hnRNPU/CTCF axis in neuroblastoma (124).

### High/low glucose regulates lncRNAs in cancer

Some studies have demonstrated that high glucose or glucose deprivation affected the expression of lncRNAs in cancer cells. For example, U87 and LN18 glioma cells after glucose deprivation had upregulation of lncRNA TP53TG1 and glucose metabolism-associated genes, including LDHA, IDH1 and GRP79 (125). Downregulation of TP53TG1 suppressed proliferation and migration of U87 cells after glucose deprivation, while overexpression of TP53TG1 displayed the opposite functions (125). Low glucose condition promoted the efficacy of TP53TG1 compared with high glucose condition. This study suggested that glucose metabolism dysregulation can affect the expression of TP53TG1 and tumor proliferation and migration in glioma (125).

High glucose increased the expression of miR-483-3p in hepatocellular carcinoma (HCC) cells. Moreover, upregulation of miR-483-3p inhibited the expression of ER protein 29 (ERp29), resulting in promotion of proliferation and migration of HCC cells (126). Furthermore, lncRNA MEG3 can bind with miR-483-3p in HCC cells. High glucose also reduced the expression of lncRNA MEG3 in HCC cells. Consistently, silencing of lncRNA MEG3 suppressed the expression of ERp29 in HCC cells (126). This study showed that high glucose could affect the expression of lncRNA MEG3 and govern the miR-483-3p/ERp29 proteins in HCC patients, suggesting that management of lncRNA MEG3 could be promising for the treatment of HCC patients with diabetes (126). Low glucose elevated the expression of lncRNA HOXC-AS3, leading to promotion of metabolic reprogramming of breast cancer *via* binding to SIRT6 and inactivating HIF1α (127).

## Targeting lncRNAs for treating DKD and cancer

Klotho is often known as an antiaging protein to prevent of aging. Klotho has been identified to protect renal tubular EMT during the DKD development (52). Overexpression of Klotho reduced the lncRNA NEAT1 expression in HFD/STZ-mediated DKD mice. Moreover, overexpression of Klotho attenuated the expression levels of NEAT1 in BSA-treated HK2 cells (52). On the contrary, knockdown of Klotho increased the expression of lncRNA NEAT1 in HK2 cells. Thereby, knockdown of Klotho caused upregulation of NEAT1 and activation of EMT and fibrosis in a ERK1/2-dependent manner (52). Another study showed that Klotho blocked EMT *via* downregulation of early growth response factor 1 (Egr-1) by suppression of the ERK1/2 pathway in DKD mice (128). Similarly, Klotho decreased Egr-1 expression *via* repressing TGF-β1/Smad3 pathway in human mesangial cells after high glucose exposures (129). Triptolide, a diterpenoid epoxide that is obtained from the thunder god vine, blocked renal tubular EMT *via* modulation of miR-188-5p-involved PI3K/Akt pathway in DKD (130). Several studies have showed that triptolide regulated the expression of multiple lncRNAs, including lncRNAs WAKMAR2, PACER, ENST00000619282, RP11-83J16.1 (131–135). Therefore, whether triptolide regulates the lncRNA expression in DKD needs to be further explored. Berberine, an isoquinoline alkaloid, has been reported to upregulate the expression of lncRNA GAS5 to reduce the mitochondrial ROS generation in HK-2 cells under high glucose environment through regulation of miR-18a-5p and C/EBPβ expression (136). The antisense oligonucleotide treatment by targeting specific lncRNAs could provide targeted medicine to cure DKD and cancer in the future.

## Conclusion

In summary, burgeoning data demonstrate that lncRNAs play an essential role in the development of DKD and diabetes-associated cancer. LncRNAs could be diagnosis and prognosis biomarkers for DKD and diabetes-related cancer. Modulation of lncRNAs might be a promising strategy for treating DKD and diabetes-associated cancer. It is important to note that it is far from being fully clarified, although some studies have explored the role of lncRNAs in DKD and cancer patients with DM. A small number of lncRNAs are identified in regulation of DKD and cancer patients with abnormal glucose metabolism. Whether other lncRNAs also participate in DKD and diabetes-associated cancer need to be explored. Compared with other factors such as m6A and signaling pathways, it remains questionable whether lncRNAs are more important in modulation of DKD and diabetes-related cancers. Addressing these questions will help us understand the mechanism of lncRNAs-regulated DKD and cancers, which could provide the clues for discovering new therapeutic strategy for DKD and cancer patients with diabetes.

## Author contributions

YC and XW wrote the manuscript. YX made the figures. YC and PW edited the manuscript and supervised this study. All authors read and approved the final manuscript.

## Funding

This work was supported by National Natural Science Foundation of China (82260876) and Hainan Province Science and Technology Special Fund (ZDKJ2021034).

## Conflict of interest

The authors declare that the research was conducted in the absence of any commercial or financial relationships that could be construed as a potential conflict of interest.

## Publisher’s note

All claims expressed in this article are solely those of the authors and do not necessarily represent those of their affiliated organizations, or those of the publisher, the editors and the reviewers. Any product that may be evaluated in this article, or claim that may be made by its manufacturer, is not guaranteed or endorsed by the publisher.

## Abbreviations

α-SMA, alpha smooth muscle actin; α1-MG, α1-microglobulin; APEX2, ascorbate peroxidase; β2-MG, β2-microglobulin; BSA, bovine serum albumin; CASC2, cancer susceptibility candidate 2; ChREBP, carbohydrate response element binding protein; CTGF, connective tissue growth factor; DRAIR, diabetes regulated anti-inflammatory lncRNA; DKD, diabetic kidney disease; HbA1c, glycosylated hemoglobin; HDAC1, histone deacetylase 1; HKDC1, hexokinase domain-containing 1; HOTAIR, HOX antisense intergenic RNA; LncRNAs, long noncoding RNAs; MEG3, maternally expressed gene 3; MLX, MAX dimerization protein; MXD1, MAX dimerization protein 1; NEAT, nuclear-enriched abundant transcript; PBMC, peripheral blood mononuclear cells; PGC-1α, peroxisome proliferator-activated receptor γ coactivator α; SOD, superoxide dismutase; TGF-β1, transforming growth factor-β1; TME, tumor microenvironment; TUG1, taurine upregulated gene 1.
